# Prevalence of *PIK3CA* mutations in Taiwanese patients with breast cancer: a retrospective next-generation sequencing database analysis

**DOI:** 10.3389/fonc.2023.1192946

**Published:** 2023-08-15

**Authors:** Ta-Chung Chao, Yi-Fang Tsai, Chun-Yu Liu, Pei-Ju Lien, Yen-Shu Lin, Chin-Jung Feng, Yen-Jen Chen, Jiun-I. Lai, Chih-Yi Hsu, Jiun Jen Lynn, Chi-Cheng Huang, Ling-Ming Tseng

**Affiliations:** ^1^ Comprehensive Breast Health Center, Department of Surgery, Taipei Veterans General Hospital, Taipei, Taiwan; ^2^ School of Medicine, College of Medicine, National Yang-Ming Chiao Tung University, Taipei, Taiwan; ^3^ Division of Cancer Prevention, Department of Oncology, Taipei Veterans General Hospital, Taipei, Taiwan; ^4^ Division of General Surgery, Department of Surgery, Taipei Veterans General Hospital, Taipei, Taiwan; ^5^ Division of Transfusion Medicine, Department of Medicine, Taipei Veterans General Hospital, Taipei, Taiwan; ^6^ Division of Medical Oncology, Department of Oncology, Taipei Veterans General Hospital, Taipei, Taiwan; ^7^ Institute of Clinical Medicine, School of Medicine, National Yang Ming Chiao Tung University, Taipei, Taiwan; ^8^ Department of Pathology and Laboratory Medicine, Taipei Veterans General Hospital, Taipei, Taiwan; ^9^ Medical Affairs, Novartis (Taiwan) Co. Ltd, Taipei, Taiwan; ^10^ Institute of Epidemiology and Preventive Medicine, College of Public Health, National Taiwan University, Taipei, Taiwan

**Keywords:** advanced breast cancer, *PIK3CA* mutations, hotspot mutations, next-generation sequencing, Taiwanese population

## Abstract

**Background:**

Breast cancer is the most common cancer type that affects women. In hormone receptor–positive (HR+), human epidermal growth factor receptor 2−negative (HER2–) advanced breast cancer (ABC), phosphatidylinositol-4,5-bisphosphate 3-kinase catalytic subunit alpha (*PIK3CA)* is the most frequently mutated gene associated with poor prognosis. This study evaluated the frequency of *PIK3CA* mutations in the Taiwanese breast cancer population.

**Methodology:**

This is a retrospective study; patient data were collected for 2 years from a next-generation sequencing database linked to electronic health records (EHRs). The primary endpoint was the regional prevalence of *PIK3CA* mutation. The secondary endpoints were to decipher the mutation types across breast cancer subtype, menopausal status, and time to treatment failure after everolimus (an mTOR inhibitor) or cyclin-dependent kinase 4/6 (CDK4/6) inhibitor treatment.

**Results:**

*PIK3CA* mutations were identified in 278 of 728 patients (38%). *PIK3CA* mutations were reported in 43% of patients with HR−/HER2+ subtype and 42% of patients with HR+/HER2– postmenopausal status. A lower prevalence of *PIK3CA* mutations was observed in triple-negative (27%) and HR+/HER2– premenopausal patients (29%). The most common mutation was at exon 20 (H1047R mutation, 41.6%), followed by exon 9 (E545K mutation, 18.9% and E542K mutation, 10.3%). Among patients treated with CDK4/6 inhibitors, the median time to treatment failure was 12 months (95% CI: 7-21 months) in the *PIK3CA* mutation cohort and 16 months (95% CI: 11-23 months) in the *PIK3CA* wild-type cohort, whereas patients receiving an mTOR inhibitor reported a median time to treatment failure of 20.5 months (95% CI: 8-33 months) in the *PIK3CA* mutation cohort and 6 months (95% CI: 2-9 months) in the *PIK3CA* wild-type cohort.

**Conclusion:**

A high frequency of *PIK3CA* mutations was detected in Taiwanese patients with breast cancer, which was consistent with previous studies. Early detection of *PIK3CA* mutations might influence therapeutic decisions, leading to better treatment outcomes.

## 1 Introduction

Breast cancer is the most commonly diagnosed cancer and one of the leading causes of cancer death among women in Taiwan. In 2019, Taiwan reported 14,856 new cases of breast cancer and 2633 deaths that occurred due to breast cancer (1). Despite the wide range of therapeutic interventions available for breast cancer, important current advances are focused on genetic profiling to allow a mutation-driven, targeted, and effective therapeutic approach.

Phosphoinositide 3-kinase (PI3K) is the most frequently disrupted signaling pathway in hormone receptor−positive (HR+) breast cancer (2). Phosphatidylinositol 3-kinase alpha (PI3Kα) is a heterodimeric protein complex composed of the catalytic subunit p110α (coded by the phosphatidylinositol-4,5-bisphosphate 3-kinase catalytic subunit alpha [*PIK3CA*] gene) and the regulatory subunit p85α (coded by the *PIK3R1* gene) (3). Mutations of the *PIK3CA* gene, inducing hyperactivation of the alpha isoform (p110α) of PI3K, occur in 28% to 46% of patients with HR+/human epidermal growth factor receptor-2–negative (HER2–) advanced breast cancer (ABC) (4, 5). This variant is associated with poor response to HER2 targeted therapy, endocrine therapy, and chemotherapy (6). The presence of an oncogenic PI3K mutation has also been correlated with a worse clinical outcome in patients with ABC receiving cyclin-dependent kinase 4/6 (CDK4/6) inhibitors (7). The main “hotspots” reported for *PIK3CA* mutations are E542K and E545K of the helical domain on exon 9 and H1047R of the kinase domain on exon 20 (8–10). Advancement in next-generation sequencing (NGS) technologies has made gene sequencing and mutation analysis feasible and effective for clinical application in breast cancer. NGS is helpful in identifying the key mutations for guiding personalized therapy (11). A study conducted by Huang et al. in Taiwanese patients with breast cancer identified *PIK3CA* as one of the most frequently mutated genes in 38% of the study population, followed by *ERBB2* (23%), *ESR1* (10%), *AKT1* (6%), and *BRCA2* (5%) mutations (12).

Alpelisib, a selective PI3K inhibitor, showed efficacy in patients with *PIK3CA*-mutated HR+/HER2– ABC in the SOLAR-1 trial, with a median progression-free survival (PFS) of 11.0 months (95% confidence interval [CI]: 7.5-14.5) (13). Based on these results, alpelisib was approved in Taiwan since December 2020 for the treatment of postmenopausal women and men, with HR+/HER2–, *PIK3CA*-mutated, locally advanced or metastatic breast cancer following progression on or after an endocrine-based regimen (14). Because the data on the prevalence of *PIK3CA* mutations in the Taiwanese population are limited, this study aimed to evaluate the frequency of *PIK3CA* mutation status, thereby determining the patient pool that might benefit from a personalized treatment plan. Here, we report the results from a single-center observational study that investigated the frequency of *PIK3CA* mutations in Taiwanese patients with all subtypes of breast cancer using NGS over a period of 24 months.

## 2 Methods

This is a retrospective, single-center study investigating the prevalence of *PIK3CA* mutations in female patients diagnosed with breast cancer in Taiwan.

### 2.1 Trial design

Taipei Veterans General Hospital, Taiwan, has an ongoing project (VGH-TAYLOR) performing comprehensive genomic profiling on tumor tissues from patients with breast cancer via NGS, which is sponsored by the YongLin Healthcare Foundation. The VGH-TAYLOR study aimed to discover potential biomarkers for recurrence, diagnosis, and prognosis of breast cancer that may enable personalized medicine and improvement in breast cancer treatment; the rationale and design of the study protocol have been previously described (15). Patient and genomic data were collected from the NGS database linked with electronic health records (EHRs) to investigate the mutation prevalence in various subtypes and stages of breast cancer.

**Figure 1:**
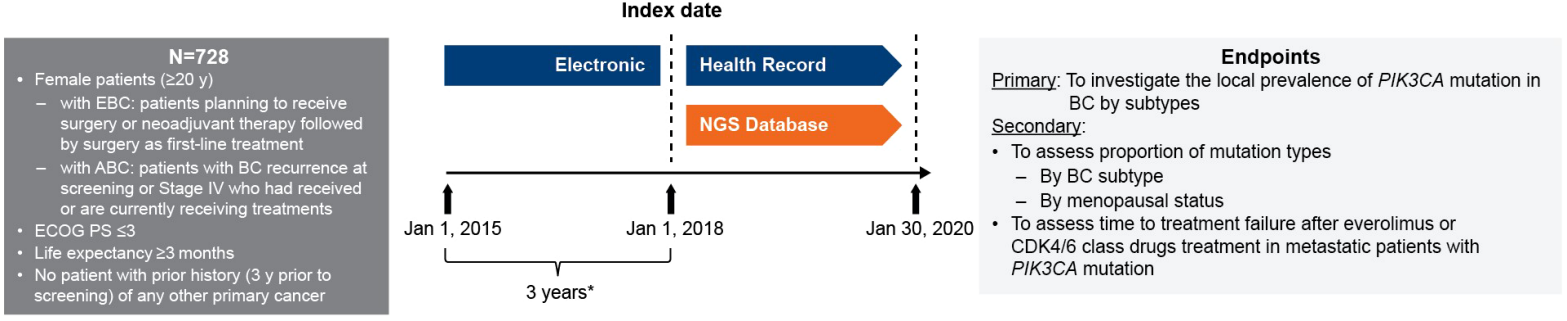
Study design. ^*^ Baseline characteristics collection period from HER. ABC, advanced breast cancer; BC, breast cancer; CDK4/6, cyclin-dependent 4 or 6 kinase; EBC, early breast cancer; ECOG PS, Eastern Cooperative Oncology Group performance status; EHC, electronic health record; NGS, next-generation sequencing; *PIK3CA*, phosphatidylinositol-4,5-bisphosphate 3-kinase catalytic subunit alpha*;* y, years.

In the NGS database, medical records of patients with advanced/metastatic breast cancer who were treated with an mTOR inhibitor or CDK4/6 inhibitor, with treatment initiation (index date) from January 1, 2018 to January 30, 2020, were included in the study once the inclusion/exclusion criteria were met. The EHRs were retrieved 3 years prior to the index date, from January 1, 2015 to January 1, 2018, for baseline characteristics and breast cancer recurrence data collection (**Figure 1**). Patients with information of first prescription of mTOR or CDK4/6 inhibitors during the 4-year EHR data collection period were also included for analysis for time to treatment failure. The study protocol was approved by the ethics committee of the Taipei Veterans General Hospital.

The pathological features of the tissue samples were determined using immunohistochemistry (IHC) assay. At least 1% of nuclei staining–positive results were defined as ER-positive. As per the American Society of Clinical Oncology (ASCO) and College of American Pathologists (CAP) guidelines, IHC 3+ and IHC 2+ with fluorescence *in situ* hybridization (FISH) amplification indicated HER2 overexpression. An IHC score of 0 to 1+ is called HER2– and a score of 3+ is called HER2+. A FISH HER2:CEP17 signal ratio >2.2 is called amplification whereas, ratio between 1.8-2.2 is equivocal and <1.8 is non-amplification

### 2.2 Patients

Inclusion criteria were defined as follows: (1) female patients aged ≥20 years; (2) patients with confirmed diagnosis of primary invasive breast cancer who are planning to receive treatment for breast cancer; (3) patients who have breast cancer recurrence at screening or Stage IV patients who have received or are currently receiving treatments for breast cancer; (4) Eastern Cooperative Oncology Group (ECOG) performance status ≤3; and (5) life expectancy ≥3 months.

Patients were excluded if they had a primary cancer other than breast cancer within 5 years prior to screening. Archival samples from the biobank (retrospective cohort) will be withdrawn if: (1) tumor content of the FFPE sample is lower than the specified percentage according to the standard of the central laboratory; (2) FFPE samples failed the DNA/RNA quality check. The criteria of the DNA/RNA quality check will follow the standard of the central laboratory. Enrolled patients will be withdrawn if one of the following conditions occurs: (1) patient withdraws consent; (2) patient refuses to provide specimens for evaluation after enrollment; (3) patient for which all samples/specimens fail the DNA/RNA quality check. The criteria of the DNA/RNA quality check will follow the standard of the central laboratory; (4) patient who does not have sufficient FFPE samples, tissues or blood samples for genetic profiling analysis by principal investigator’s discretion; (5) patient who does not return to the clinical site for more than 6 months (based on their medical records) will be considered as lost to follow-up. However, whether this subject should be withdrawn will be based on the PI’s discretion (15).

**Figure 2:**
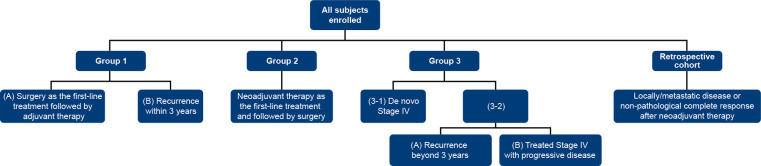
Patient distribution.

In this study we retrospectively analyze the database from VGH-TAILOR project (Group 1,2,3). Enrolled patients were categorized into 4 major groups, presented in **Figure 2**.

#### 2.2.1 Group 1

• Group 1A (early breast cancer): patients who were planning to receive surgery as the first-line treatment followed by adjuvant therapy.

• Group1B (advanced breast cancer): patients with recurrence at screening, who had received surgery for primary breast cancer within 3 years prior to screening, and with primary tumor formalin-fixed paraffin-embedded (FFPE) tissues available.

#### 2.2.2 Group 2 (early breast cancer)

Patients who were planning to receive neoadjuvant therapy as the first-line treatment for breast cancer and followed by surgery.

#### 2.2.3 Group 3 (advanced breast cancer)

• Group 3-1: patients diagnosed with *de novo* and treatment naïve stage IV breast cancer.

• Group 3-2: patients diagnosed with a stage IV breast cancer and with recurrence beyond 3 years after surgery (Group 3-2A) or stage IV subjects who had received or are currently receiving treatments for breast cancer (Group 3-2B).

#### 2.2.4 Retrospective cohort

Samples were collected from the biobank of the patients with local/metastatic disease or non-pathological complete response after neoadjuvant therapy

### 2.3 Endpoints

The primary endpoint was the regional prevalence of *PIK3CA* mutation in breast cancer and by breast cancer subtypes in overall patients with breast cancer, patients with HR+/HER2–, HR−/HER2+, HR+/HER2+ and triple-negative breast cancer. Because the pre- and postmenopausal status present different biological and genetic characteristics in HR+/HER2– breast cancer, the prevalence of *PIK3CA* mutations was further analyzed by menopausal status in HR+/HER2– breast cancer. Menopause is usually a clinical diagnosis made after ≥12 months of amenorrhea (16).

Secondary endpoints were to assess the frequency of mutation types by breast cancer subtype and by menopausal status and time to treatment failure after mTOR or CDK4/6 inhibitor in metastatic patients stratified by *PIK3CA* mutation status. Time to treatment failure is defined as the time from the date of first dose of treatment of interest to the date of the patient discontinuing treatment for any reason; the Kaplan-Meier (KM) method was used for the estimation.

### 2.4 Sample preparation

The genetic profiles were determined through NGS of FFPE samples. For screening and recruitment of patients for Group 1B and Group 3-2, paired FFPE primary and recurrent tumor samples were collected and sequenced, and for Group 2, paired FFPE diagnostic and post-neoadjuvant specimens were assayed.

The preparation of FFPE was done under standard conditions at the trial site. The DNA/RNA extraction and hematoxylin and eosin (H&E) staining were performed in accordance with the laboratory manual under the guidance of a certified pathologist in the central laboratory. Quality checks for DNA/RNA were performed as per the manual of the Thermo Fisher™ Oncomine™ (TMO) Comprehensive Assay requirement (see below) and additional samples were collected in case of failure. Of the seven unstained FFPE sections retrieved (per subject), one section was prepared for H&E staining and six sections were prepared for TMO comprehensive assay.

### 2.5 Oncomine™ comprehensive assay (TMO comprehensive assay)

Oncomine™ comprehensive assay (TMO comprehensive assay, Thermo Fisher Scientific, Waltham, MA) was used to profile thousands of variants across 161 cancer-relevant genes using FFPE tissues (17). Analyses of TMO comprehensive assay included identification of genes and detection of mutation types such as frameshift, missense, synonymous, single nucleotide variation (SNV), insertion/deletion (Indel), and copy number variation (CNV) observed in individual subject.

Amplicon libraries were constructed with multiplex polymerase chain reaction (PCR) primers for preparation of DNA and RNA (for fusion genes) from FFPE samples. Sequencing was performed with the Ion Gene Studio S5 System and Ion 540 Chips. Raw data process, alignment, and variant calling were performed with Torrent Suite™ Software, with variant calling using the Torrent Variant Caller plug-in. Further management was proceeded by Ion Reporter™ Software with workflow “Oncomine Comprehensive v3 - w3.2 - DNA and Fusions - Single Sample” version 5.10 selected and filter chain “Oncomine Variants” version 5.10 applied. Reference genome was hg19.

### 2.6 Variables

Preindex variables, including demographics of all patients, menopausal status, primary diagnosis, previous medical history/comorbidities, and family history of cancer, were extracted from the NGS database, whereas the treatment response of previous breast cancer was taken from the EHRs.

Postindex variables in the follow-up period, including breast cancer genetic mutations, treatment, and treatment response from previous lines if any, were extracted from the NGS database, and breast cancer recurrence data were also extracted from the EHRs.

### 2.7 Statistical analysis

All statistical tests were conducted with a two-sided alternative hypothesis, and a significance level of 0.05 and a *P* value of <0.05 were considered statistically significant, unless otherwise specified. Mutations in the PIK3/AKT/mTOR pathway were deciphered. The prevalence of *PIK3CA* mutations was calculated as the number of *PIK3CA* mutations divided by the number of patients with breast cancer in the populations stated above. The 95% CIs were presented as appropriate. Analysis of the primary endpoint was descriptive in nature, and no statistical hypothesis or testing was performed. The enrolled set was adopted for both primary and secondary endpoints unless otherwise specified.

## 3 Results

### 3.1 Patient demographics and baseline characteristics

A total of 728 patients were included for the *PIK3CA* mutation analysis, most of whom were categorized into Group 1A (481 patients, 66.07%), followed by Group 2 (93 patients, 12.77%), Group 3-2 (59 patients, 8.10%), Group 3-1 (42 patients, 5.77%), and Group 1B (20 patients, 2.75%), please refer to **Figure 2** for what each group stands for. The mean age in all the groups at the time of testing was 51 to 57 years of age. A total of 548 patients (76.2%) with HR+ and 149 patients (20.8%) with HER2+ status were reported. Patient demographic characteristics are detailed in **Table 1**.

**Table 1:** Patient demographic characteristics (excluding retrospective cohort).

Group	Group 1A	Group 1B	Group 2	Group 3-1	Group 3-2
**Case number**	481	20	93	42	59
**Age, mean** (SD)	57 (12)	56 (13)	51 (12)	57 (11)	55 (12)
**Age** (min-max)	22-93	34-80	27-81	25-83	31-83
**Estrogen receptor** Positive : Negative Missing	388:91 2	11:8 1	55:38 0	27:15 0	41:13 4
**HER2** Positive : Equivocal (IHC2+):Negative Missing	72:6:400 3	7:1:10 2	31:0:62 0	16:1:25 0	15:1:35 8
Stage					
I	182	5	1	0	4
II	289	9	82	0	11
III	0	0	10	0	0
IV	0	0	0	42	37
Missing	10	6	0	0	7
Grade					
I	83	0	6	2	2
II	256	9	68	30	22
III	135	7	18	10	17
Missing	7	4	1	0	18

Staging of group 1B and 3-2 indicated original stages of primary tumor.

HER2 Positive: IHC2+: Negative: Missing → Positive: Equivocal: Negative: Missing

Equivocal: HER2 immunohistochemical stain score 2+ without in situ hybridization testing; IHC2: without FISH testing.

FISH, fluorescence in situ hybridization; HER2, human epidermal growth factor receptor 2; IHC, immunohistochemistry; SD, standard deviation.

### 3.2 Prevalence and characteristics of *PIK3CA* mutations

**Figure 3:**
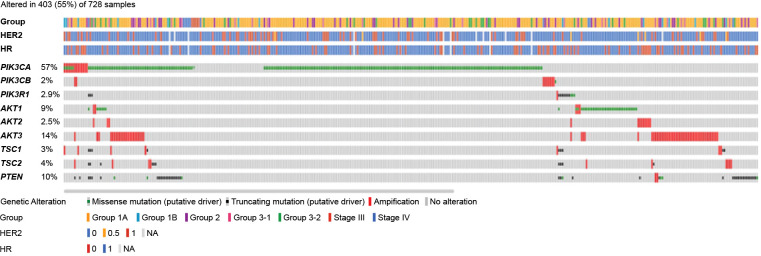
Mutation map of genes in the PI3K/AKT/mTOR pathway. *AKT*, serine/threonine kinase*;* HR, hormone receptor; HER2, human epidermal growth factor receptor 2; mTOR, mammalian target of rapamycin; PI3K, phosphoinositide 3-kinase; *PIK3CA/PIK3CB*, phosphatidylinositol-4,5-bisphosphate 3-kinase catalytic subunit alpha/beta; *PIK3R1*, phosphoinositide-3-kinase regulatory subunit 1; *PTEN*, phosphatase and TENsin homolog; *TSC*, tuberous sclerosis complex.

Overall, 403 of 728 patients (55%) had mutations in the PI3K/AKT/mTOR pathway, of which the 3 most commonly detected mutations were *PIK3CA* (57%), *AKT3* (14%), and *PTEN* (10%) (**Figure 3**).

**Figure 4:**
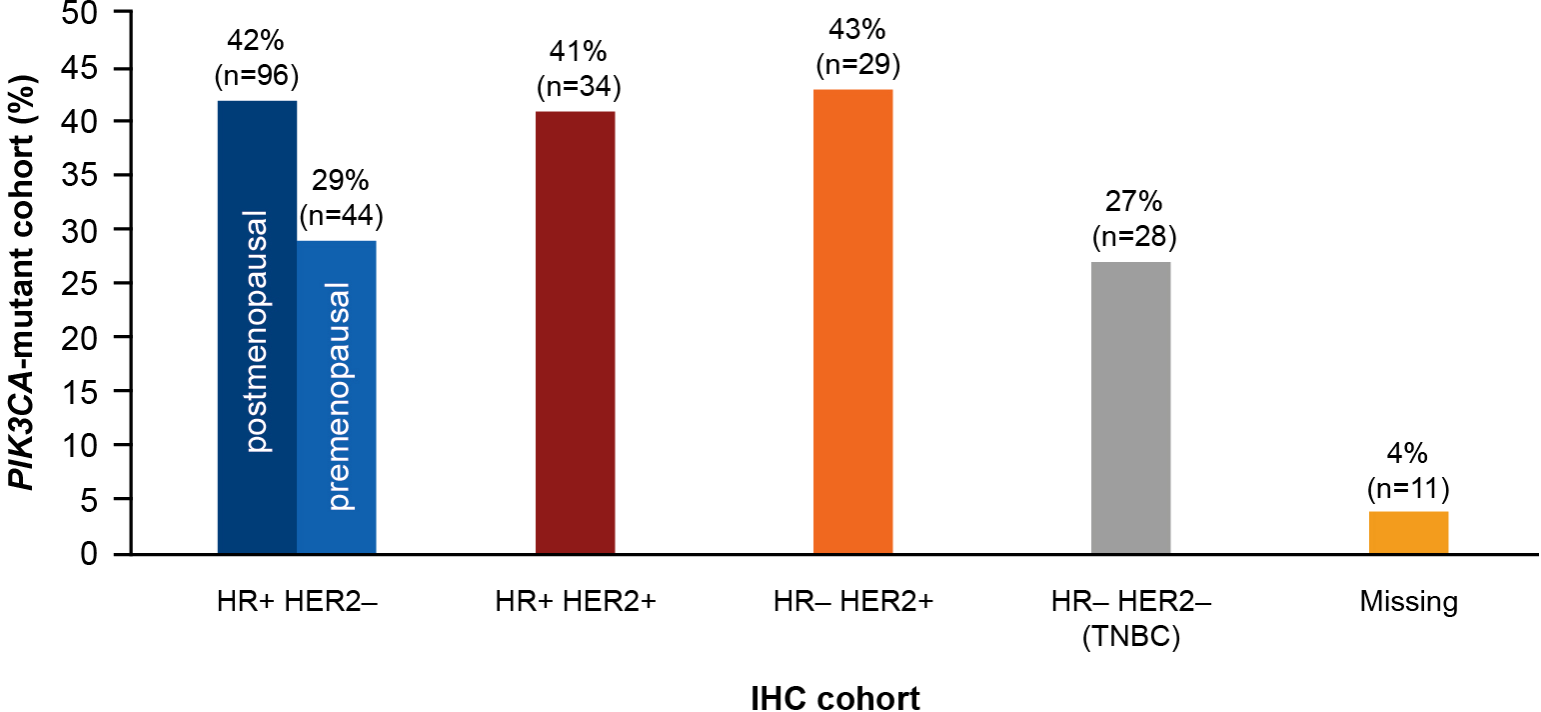
Prevalence of *PIK3CA* mutation in each IHC cohort. HR, hormone receptor; HER2, human epidermal growth factor receptor 2; IHC, immunohistochemistry; *PIK3CA*, phosphatidylinositol-4,5-bisphosphate 3-kinase catalytic subunit alpha; TNBC, triple-negative breast cancer.

A total of 278 of 728 patients (38%) harbored *PIK3CA* mutations. With respect to the IHC phenotypes, 29 patients (43.0%) were HR−/HER2+, 96 patients (42.0%) were HR+/HER2– (postmenopausal), and 34 patients (41.0%) were HR+/HER2+. The prevalence of *PIK3CA* mutation was relatively lower in triple-negative breast cancer (27.0%) and premenopausal patients with HR+/HER2– status (29.0%). The prevalence of *PIK3CA* mutation by IHC status is presented in **Figure 4**.

**Figure 5:**
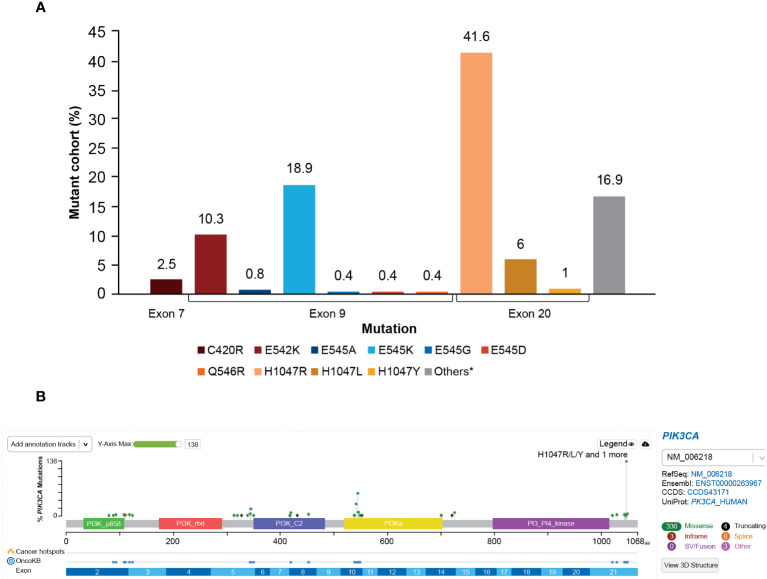
**(A)** Distribution of *PIK3CA* variants frequency. *A total of 16.9% of mutations in patients from this study were not captured by the therascreen *PIK3CA* Rotor-Gene Q (RGQ) PCR Kit. The most frequent mutations not captured were E726K (2.8%) and N345K (5.3%). Mutation Q546E was not detected (0%) at exon 9. **(B)** OncoPrinter plot of *PIK3CA* mutations. PI3K, phosphoinositide 3-kinase; *PIK3CA*, phosphatidylinositol-4,5-bisphosphate 3-kinase catalytic subunit alpha.

The majority of the *PIK3CA* mutations were clustered in exon 9 and exon 20, helical and kinase domains, respectively. Among 278 patients with *PIK3CA* mutations, the most frequently observed mutations were in exon 20 (H1047R, 41.6%) and exon 9 (E545K, 18.9% and E542K, 10.3%); these are presented in **Figure 5A**.

The distribution of *PIK3CA* mutation types is presented in **Figure 5B**. A total of 27 of 278 patients (9.7%) harbored multiple *PIK3CA* mutations within one sample. Mutual exclusivity was observed between *PIK3CA* and *AKT1* (*P*<0.001), *PIK3CA* and *AKT3* (*P*<0.001), and *PIK3CA* and *PIK3R1* (*P*=0.007).

### 3.3 *PIK3CA* mutations in patients with HR+/HER2– breast cancer by menopausal status

*PIK3CA* hotspot mutations (H1047R, E545K, and E542K) were found in 63.5% of premenopausal patients with breast cancer and in 56.2% of postmenopausal patients with HR+/HER2– breast cancer. Among the premenopausal *PIK3CA-*mutated patients, H1047R and E545K mutations were the most common (27% each), followed by the E542K mutation (9.5%). A similar distribution was seen in postmenopausal patients, where the H1047R mutation was the most common mutation (32%), followed by E545K (14.5%) and E542K mutations (9.7%).

### 3.4 Time to treatment failure in patients with *PIK3CA* mutation after CDK4/6 inhibitors or mTOR inhibitor

**Figure 6:**
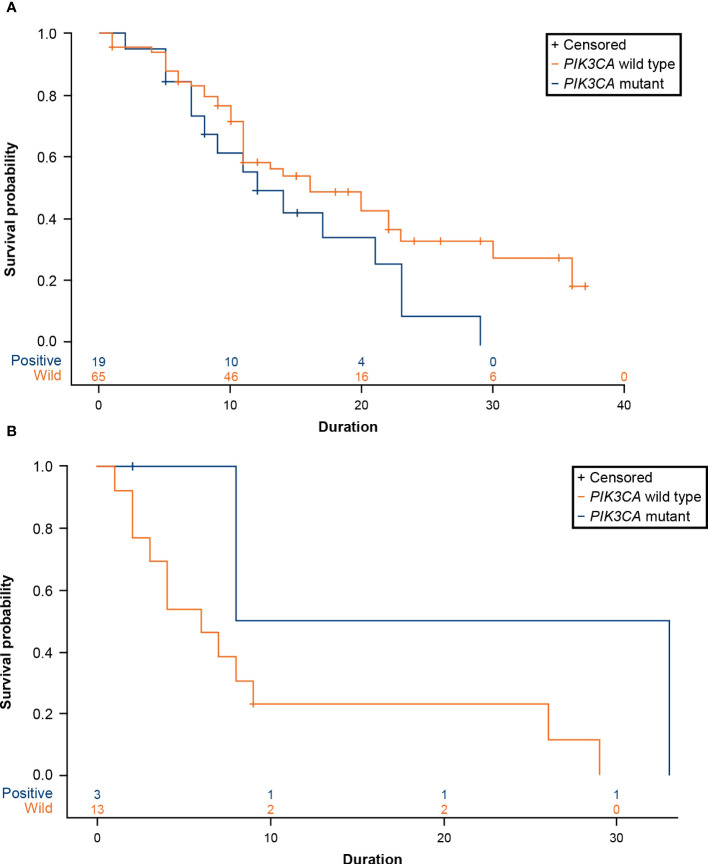
**(A)** Product-limit survival estimates of CDK4/6 inhibitors. **(B)** Product-limit survival estimates of everolimus (an mTOR inhibitor). CDK4/6, cyclin-dependent 4 and 6 kinase; mTOR, mammalian target of rapamycin; *PIK3CA*, phosphatidylinositol-4,5-bisphosphate 3-kinase catalytic subunit alpha.

A total of 19 patients (22.62%) with advanced breast cancer who received CDK4/6 inhibitors (n=84, ribociclib=47, palbociclib=32, abemaciclib=5) and 3 patients (18.75%) who received an mTOR inhibitor (n=16) were reported to have a *PIK3CA* mutation. For patients treated with CDK4/6 inhibitors, the median time to treatment failure was 16 months (95% CI: 11-23 months) in the *PIK3CA* wild-type cohort and 12 months (95% CI: 7-21 months) in the *PIK3CA* mutation cohort, with a hazard ratio (HR) of 1.670 (95% CI: 0.908-3.069) (**Figure 6A**; **Table 2**). The data demonstrated a trend of shorter treatment duration with CDK4/6 inhibitors in patients with *PIK3CA* mutation. For patients receiving mTOR inhibitor, the median time to treatment failure was 20.5 months (95% CI: 8-33 months) in the *PIK3CA* mutation cohort and 6 months (95% CI: 2-9 months) in the *PIK3CA* wild-type cohort, with an HR of 0.244 (95% CI: 0.031-1.922) (**Figure 6B**; **Table 2**).

**Table 2:** CDK4/6 and mTOR inhibitors on patients with *PIK3CA* mutation*.

Drug	Patient number	Median number of lines of therapy	Median time to treatment failure (months)		Hazard ratio, 95% CI
			*PIK3CA* mutant	*PIK3CA* wild type	
CDK4/6 inhibitors	84	1 (1-5)	12 (n=19, 95% CI: 7-21)	16 (n=65, 95% CI: 11-23)	1.670, 0.908-3.069
mTOR inhibitor (Everolimus)	16	2 (1-6)	20.5 (n=3, 95% CI: 8-33)	6 (n=13, 95% CI: 2-9)	0.244, 0.031-1.922

*Patients with treatment exposure of <1 month were excluded.

CDK4/6, cyclin-dependent 4 and 6 kinase; CI, confidence interval; mTOR, mammalian target of rapamycin; *PIK3CA*, phosphatidylinositol-4,5-bisphosphate 3-kinase catalytic subunit alpha.

## 4 Discussion

*PIK3CA* mutations are significantly associated with breast cancer occurrence and provide a growth advantage to cancer cells, which leads to progression and drug resistance (18). Hence, early detection of *PIK3CA* mutations may help in identifying patients who might benefit from a more effective personalized targeted therapy (19). In the present study, we reported the prevalence of *PIK3CA* mutations in Taiwanese patients with breast cancer stratified by IHC subtypes using an NGS database. In addition, we assessed the impact of *PIK3CA* mutation status on time to treatment failure of CDK4/6 and mTOR inhibitors in patients with metastatic breast cancer.

In this study, genes in PIK3/AKT/mTOR pathway were deciphered, and *PIK3CA* mutations were identified as the most frequent genetic alterations observed in 38% of Taiwanese patients with breast cancer. Similarly, an earlier study by Huang et al. reported *PIK3CA* mutation as the most frequent mutation occurring in 38% of Taiwanese patients with breast cancer (12). The differences in the *PIK3CA* mutation frequency observed between the 2 studies could be attributed to the different sampling sizes and techniques of analysis.

The frequency of *PIK3CA* mutations varied by breast cancer subtype and menopausal status. A high prevalence of *PIK3CA* mutations was observed in patients with HR−/HER2+ status (43%) and HR+/HER2– postmenopausal status (42%), followed by HR+/HER2+ patients (41%). A comparatively lower prevalence was seen in HR+/HER2– premenopausal patients (29%), followed by patients with triple-negative breast cancer (27%). Although there is no consensus about a predisposition of *PIK3CA* mutation by breast cancer subtype, several studies have reported that 13.3% to 61.5% of HR+/HER2– ABC tumors and 12% to 25% of HER2+ tumors had *PIK3CA* mutations, whereas triple-negative breast cancer harbors the lowest rates of *PI3KCA* mutations (20, 21). A recent-published study (AURORA, BIG 14-01) analyzed 339 patients treated with first-line endocrine therapy plus CDK4/6 inhibitor, and found that *PIK3CA* mutation rate was 40%, which was similar to our findings (42% in HR+/HER2– patients). These mutations were not associated with first-line outcome (HR: 1.07 [95% CI: 0.66-1.75], *P*=0.65) (22). In our study with limited case numbers (n=84) and heterogenous characteristics (CDK4/6 inhibitors not purely first-line), it is difficult to compare the outcome in patients with *PIK3CA* mutations in these two studies. In our study, the most commonly reported hotspot mutations were H1047R (41.6%), followed by E545K (18.9%) and E542K (10.3%). These results were consistent with prior findings that reported H1047R, E545K, and E542K as the most common hotspots for *PIK3CA* mutations (5, 8, 9).

In the present study, 27 patients were reported with multiple *PIK3CA* mutations. Of note, a prior report by Vasan et al. indicated markedly increased sensitivity of multiple *PIK3CA*-mutant tumors to PI3K inhibitors, compared with a single hotspot mutation (23). In the Taiwanese population, *PIK3CA* and *PIK3R1* mutations were observed to be mutually exclusive. This mutual exclusivity of *PIK3CA* and *PIK3R1* mutations was reported earlier, leading to oncogenesis and activation of the PI3K pathway (24).

In a recent study by Pavithran et al., the presence of *PIK3CA* mutations was associated with reduced sensitivity to CDK4/6 inhibitors (25). In the present study, the median time to treatment failure of CDK4/6 inhibitors was much lower in patients with *PIK3CA* mutations than those without *PIK3CA* mutations (12 months vs 16 months; HR: 1.670; 95% CI: 0.908-3.069). However, this profile was shorter than that in another study that reported a time to treatment failure of 19.7 months where they used CDK4/6 inhibitors as the first-line therapy (26). This difference may be attributed to the discrepancy in patient characteristics, follow-up period, or sample size (24). Findings from the MONALEESA-7 study evaluating ribociclib in premenopausal or perimenopausal women with HR+/HER2– ABC showed a shorter PFS in patients with *PIK3CA* mutations than those without, although this difference was not statistically significant (27). Collectively, multiple studies demonstrated numerical trends towards shorter PFS in patients with *PIK3CA* mutations, which might indicate that the presence of *PIK3CA* mutations may influence the sensitivity to CDK4/6 inhibitors.

Mutations in *PIK3CA* often result in hyperactivation of the PI3K/mammalian target of rapamycin (mTOR) pathway and may predict response to mTOR inhibitors. In the present study, the median time to treatment failure of an mTOR inhibitor (everolimus) was comparably longer in patients with *PIK3CA* mutations than those without (20.5 months vs 6 months; HR: 0.244; 95% CI: 0.031-1.922). Further research is required to validate these findings owing to the small sample size of the *PIK3CA-*mutant cohort (n=3). A combined analysis from BOLERO-1 and BOLERO-3 suggested that patients having tumors with *PIK3CA* mutations or hyperactive PI3K pathway derived PFS benefit from everolimus, whereas patients having tumors without these molecular alterations did not (28). Moynahan et al. reported that this survival benefit with everolimus was maintained irrespective of the type of *PIK3CA* genotype in BOLERO-2 (29). This further supports the findings of the current study. The earlier detection of *PIK3CA* mutations may help oncologists in treatment decisions and thereby help in providing an effective personalized targeted therapy to the patients.

The present study has a few limitations that are worth noting. This was a retrospective, single-center study and was prone to selection bias. Therefore, the actual frequency of genetic alterations observed in the study may not fully represent the general population in Taiwan. Lastly, the small sample size of patients receiving targeted therapies warrants further studies to validate these findings, especially in the case of time to treatment failure.

## 5 Conclusion

Consistent with previous studies, we identified a high prevalence of *PIK3CA* mutations in 38% of the Taiwanese patients with breast cancer (12). The lower prevalence in premenopausal patients and patients with triple-negative breast cancer warrants further studies. Most of the mutations were in exon 9 and exon 20, with H1047R, E545K, and E542K being the hotspots. A longer time to treatment failure in wild-type *PIK3CA* cohorts treated with CDK4/6 inhibitors was reported, which demonstrated the better efficacy of CDK4/6 inhibitors in wild-type *PIK3CA* cohorts than that in the *PIK3CA*-mutant cohort. Everolimus, an mTOR inhibitor, reported a longer time to treatment failure in the *PIK3CA-*mutant cohort and demonstrated better efficacy. Cumulatively, this indicated variations in the prevalence of *PIK3CA* mutation based on breast cancer IHC phenotype. Detection of mutations at an earlier stage can help in making appropriate therapeutic decisions, thus saving time and resulting in better outcomes for the Taiwanese breast cancer population.

## Data availability statement

The original contributions presented in the study are included in the article/supplementary material. Further inquiries can be directed to the corresponding authors.

## Ethics statement

The studies involving humans were approved by ethics committee of the Taipei Veterans General Hospital. The studies were conducted in accordance with the local legislation and institutional requirements. The participants provided their written informed consent to participate in this study.

## Author contributions

All authors critically reviewed, provided feedback at each stage of the manuscript, approved the final version and agreed to be accountable for all aspects of the work.

## References

[1] Shih N-C Kung P-T Kuo W-Y Tsai W-C. Association of treatment delay and stage with mortality in breast cancer: a nationwide cohort study in Taiwan. Sci Rep (2022) 12(1):18915. doi: 10.1038/s41598-022-23683-y 36344740 PMC9640724

[2] Hosford SR Miller TW. Clinical potential of novel therapeutic targets in breast cancer: CDK4/6, Src, JAK/STAT, PARP, HDAC, and PI3K/AKT/mTOR pathways. Pharmgenomics Pers Med (2014) 7:203–15. doi: 10.2147/PGPM.S52762 PMC4157397 25206307

[3] Vasan N Toska E Scaltriti M. Overview of the relevance of PI3K pathway in HR-positive breast cancer. Ann Oncol (2019) 30(Suppl_10):x3–x11. doi: 10.1093/annonc/mdz281 31859348 PMC6923788

[4] André F Ciruelos EM Juric D Loibl S Campone M Mayer IA. Alpelisib plus fulvestrant for PIK3CA-mutated, hormone receptor-positive, human epidermal growth factor receptor-2-negative advanced breast cancer: final overall survival results from SOLAR-1. Ann Oncol (2021) 32(2):208–17. doi: 10.1016/j.annonc.2020.11.011 33246021

[5] Martínez-Sáez O Chic N Pascual T Adamo B Vidal M González-Farré B. Frequency and spectrum of PIK3CA somatic mutations in breast cancer. Breast Cancer Res (2020) 22(1):45. doi: 10.1186/s13058-020-01284-9 32404150 PMC7222307

[6] Rodon J Dienstmann R Serra V Tabernero J. Development of PI3K inhibitors: lessons learned from early clinical trials. Nat Rev Clin Oncol (2013) 10(3):143–53. doi: 10.1038/nrclinonc.2013.10 23400000

[7] Del Re M Crucitta S Lorenzini G De Angelis C Diodati L Cavallero D. PI3K mutations detected in liquid biopsy are associated to reduced sensitivity to CDK4/6 inhibitors in metastatic breast cancer patients. Pharmacol Res (2021) 163:105241. doi: 10.1016/j.phrs.2020.105241 33049397

[8] Zhao L Vogt PK. Helical domain and kinase domain mutations in p110α of phosphatidylinositol 3-kinase induce gain of function by different mechanisms. Proc Natl Acad Sci (2008) 105(7):2652–7. doi: 10.1073/pnas.0712169105 PMC2268191 18268322

[9] Mangone FR Bobrovnitchaia IG Salaorni S Manuli E Nagai MA. PIK3CA exon 20 mutations are associated with poor prognosis in breast cancer patients. Clinics (Sao Paulo) (2012) 67(11):1285–90. doi: 10.6061/clinics/2012(11)11 PMC3488987 23184205

[10] Kang S Bader AG Vogt PK. Phosphatidylinositol 3-kinase mutations identified in human cancer are oncogenic. Proc Natl Acad Sci USA (2005) 102(3):802–7. doi: 10.1073/pnas.0408864102 PMC545580 15647370

[11] Tripathy D Harnden K Blackwell K Robson M. Next generation sequencing and tumor mutation profiling: are we ready for routine use in the oncology clinic? BMC Med (2014) 12(1):140. doi: 10.1186/s12916-014-0140-3 25286031 PMC4244054

[12] Huang C-C Tsai Y-F Liu C-Y Chao T-C Lien P-J Lin Y-S. Comprehensive molecular profiling of Taiwanese breast cancers revealed potential therapeutic targets: prevalence of actionable mutations among 380 targeted sequencing analyses. BMC Cancer (2021) 21(1):199. doi: 10.1186/s12885-021-07931-4 33632156 PMC7908797

[13] André F Ciruelos E Rubovszky G Campone M Loibl S Rugo HS. Alpelisib for PIK3CA-mutated, hormone receptor–positive advanced breast cancer. New Engl J Med (2019) 380(20):1929–40. doi: 10.1056/NEJMoa1813904 31091374

[14] Taiwan Food and Drug Administration: Assessment Report . Available at: https://www.google.com/url?sa=t&rct=j&q=&esrc=s&source=web&cd=&ved=2ahUKEwiUytLHwtL5AhXqglYBHbAEBDwQFnoECCIQAQ&url=https%3A%2F%2Fwww.fda.gov.tw%2Ftc%2Fincludes%2FGetFile.ashx%3Fmid%3D189%26id%3D36084%26t%3Ds&usg=AOvVaw1atEjQQExVG92i10vpWwob (Accessed August 19, 2022).

[15] Liu CY Huang CC Tsai YF Chao TC Lien PJ Lin YS. VGH-TAYLOR: Comprehensive precision medicine study protocol on the heterogeneity of Taiwanese breast cancer patients. Future Oncol (2021) 17(31):4057–69 . doi: 10.2217/fon-2021-0131 34665002

[16] National Comprehensive Cancer Network. Breast Cancer (Version 1.2023) (2023). Available at: https://www.nccn.org/store/Login/Register.aspx.

[17] Hovelson DH McDaniel AS Cani AK Johnson B Rhodes K Williams PD. Development and validation of a scalable next-generation sequencing system for assessing relevant somatic variants in solid tumors. Neoplasia (2015) 17(4):385–99. doi: 10.1016/j.neo.2015.03.004 PMC4415141 25925381

[18] Fusco N Malapelle U Fassan M Marchiò C Buglioni S Zupo S. PIK3CA mutations as a molecular target for hormone receptor-positive, HER2-negative metastatic breast cancer. Front Oncol (2021) 11. doi: 10.3389/fonc.2021.644737 PMC8027489 33842357

[19] Ferreira-Gonzalez A. Plasma PIK3CA mutation testing in advanced breast cancer patients for personalized medicine: A value proposition. J Appl Lab Med (2020) 5(5):1076–89. doi: 10.1093/jalm/jfaa117 32901282

[20] Anderson EJ Mollon LE Dean JL Warholak TL Aizer A Platt EA. A systematic review of the prevalence and diagnostic workup of PIK3CA mutations in HR+/HER2- metastatic breast cancer. Int J Breast Cancer (2020) 2020:3759179. doi: 10.1155/2020/3759179 32637176 PMC7322582

[21] Castaneda CA Lopez-Ilasaca M Pinto JA Chirinos-Arias M Doimi F Neciosup SP. PIK3CA mutations in Peruvian patients with HER2-amplified and triple negative non-metastatic breast cancers. Hematology/Oncol Stem Cell Ther (2014) 7(4):142–8. doi: 10.1016/j.hemonc.2014.09.007 25467032

[22] Sotiriou C Ignatiadis M Crestani TA Venet D Tyekucheva S Irrthum A. Clinico-molecular characteristics associated with outcomes in breast cancer patients treated with CDK4/6 inhibitors: Results from the AURORA Molecular Screening Initiative. J Clin Oncol (2023) 41(Suppl 16):1019. doi: 10.1200/JCO.2023.41.16_suppl.1019

[23] Vasan N Razavi P Johnson JL Shao H Shah H Antoine A. Double PIK3CA mutations in cis increase oncogenicity and sensitivity to PI3Kα inhibitors. Science (2019) 366(6466):714–23. doi: 10.1126/science.aaw9032 PMC7173400 31699932

[24] Huang CS Liu CY Lu TP Huang CJ Chiu JH Tseng LM. Targeted sequencing of Taiwanese breast cancer with risk stratification by the concurrent genes signature: A feasibility study. J Pers Med (2021) 11(7):1–9. doi: 10.3390/jpm11070613 PMC8306786 34203389

[25] Pavithran K Jayamohanan H Jose WM Soman S Vijaykumar DK Ariyannur PS. PI3K mutation is associated with reduced sensitivity to CDK4/6 inhibitors in metastatic breast cancer. Ann Oncol (2021) 32(5):S457–515. doi: 10.1016/j.annonc.2021.08.539

[26] Giridhar KV Choong GM Leon-Ferre RA O’Sullivan CC Ruddy KJ Haddad TC. Abstract P6-18-09: Clinical management of metastatic breast cancer (MBC) after CDK 4/6 inhibitors: A retrospective single-institution study. Poster Session Abstracts (2019) 79(Suppl 4):P6–18–09. doi: 10.1158/1538-7445.SABCS18-P6-18-09 PMC9829187 36045271

[27] Bardia A Su F Solovieff N Im S-A Sohn J Lee KS. Genomic profiling of premenopausal HR+ and HER2– metastatic breast cancer by circulating tumor DNA and association of genetic alterations with therapeutic response to endocrine therapy and ribociclib. JCO Precis Oncol (2021) 5:1408–20. doi: 10.1200/PO.20.00445 PMC8423397 34504990

[28] André F Hurvitz S Fasolo A Tseng L-M Jerusalem G Wilks S. Molecular alterations and everolimus efficacy in human epidermal growth factor receptor 2–overexpressing metastatic breast cancers: combined exploratory biomarker analysis from BOLERO-1 and BOLERO-3. J Clin Oncol (2016) 34(18):2115–24. doi: 10.1200/JCO.2015.63.9161 27091708

[29] Moynahan ME Chen D He W Sung P Samoila A You D. Correlation between PIK3CA mutations in cell-free DNA and everolimus efficacy in HR+, HER2– advanced breast cancer: results from BOLERO-2. Br J Cancer (2017) 116(6):726–30. doi: 10.1038/bjc.2017.25 PMC5355930 28183140

